# Assessment of symptomatically deviated nasal septum among Indian patients

**DOI:** 10.6026/9732063002001374

**Published:** 2024-10-31

**Authors:** Harshvardhan Anil Patil, Abhay D. Havle

**Affiliations:** 1 Department of ENT, Krishna Institute of Medical Sciences, Karad - 415110, Maharashtra, India; 2 Department of otorhinolaryngology, Krishna Institute of Medical Sciences, Karad - 415 539, Maharashtra, India

**Keywords:** Nasal inspiratory peak flow (NIPF), sinonasal outcome test-22 (SNOT-22), pre-operative, specificity, questionnaire, rhinologic issues, general health, clinical, radiological examination

## Abstract

Nasal Inspiratory Peak Flow (NIPF) is an easy, reliable, inexpensive, and easy-to-measure objective test with a good specificity to measure changes in nasal obstruction on the other hand, sinonasal outcome test-22 (SNOT-22) is questionnaire designed for general health and rhinologic issues. Henceforth, the aim was to evaluate the link between NIPF and SNOT-22 scores before and after surgery. 34 patients underwent clinical & radiological examination followed by questionnaire study before and after the surgery. We found that, no significant change between NIPF & SNOT-22 scores for both age and gender aspect. Thus, pre-op SNOT-22 evaluation can be made a routine exercise as it is equally reliable and highly cost effective as compared to NIPF.

## Background:

According to a study nasal blockage is a prevalent symptom in the field of rhinology [1]. Therefore, the major cause for this symptom is a deviated nasal septum [1]. It leads to outward nasal malformation, snoring and mouth breathing. Additionally, it affects the nasal cavity's airflow dynamics and the aeration of the para-nasal sinuses, leading to sinusitis. The nasal mucosa on the concave side often exhibits compensatory hypertrophy as a result of airflow alterations [2, 3-4]. A study has shown that, between 19 and 65% of people has nasal septum deviation [5]. The surgical correction of the septum, often known as septoplasty, is the treatment for the deviated nasal septum (DNS). Although surgical correction may not be necessary for some individuals with small abnormalities, it is necessary when there are nasal obstruction symptoms that are troublesome. Therefore, it is essential to assess both the subjective and objective results prior to choosing surgical treatment. As a result of the lack of a definitive agreement about the success of septoplasty, rhinologists are encountering difficulties in relation to patient satisfaction after surgical procedures [6]. The effects of treatment after chronic rhino sinusitis or DNS are being assessed using a variety of objective tests and subjective evaluation questionnaires that have developed over the years. The SNOT-22 is a widely used patient-acceptable outcome scale that is used to evaluate the severity of nasal symptoms. This is a straight-forward, well- tested questionnaire tailored especially for Sino nasal functions [7]. The SNOT-22 is a questionnaire that was developed with the purpose of measuring particular illness outcomes that combine general health concerns with rhinologic difficulties. The SNOT has a number of questions that indicate how nasal illness affects Quality of Life (QoL). These items include functional limits, psychological consequences, and physical concerns. Anderson and his colleagues were the ones who first proposed it in the year 1998 [8]. There have been a number of studies published in the scientific literature on the use of SNOT-22 in the treatment of chronic rhinosinusitis. However, there are only a few studies that come from the Indian subcontinent, and its usage in patients who have had septoplasty has not been well explored. Therefore, it is of interest to report a correlation between the NIPF scores and the changes in the SNOT-22 scores both before and after surgery.

## Materials and Methods:

The current Observational Cohort study was conducted in the E.N.T OPD at KIMS, Karad a total of 18 months starting from August 2022 To January 2024 with 34 patients in total after getting the ethical approval and informed consent respectively. All patients underwent a detailed clinical examination which included diagnostic nasal endoscopy (N-ED) and X-RAY paranasal sinuses (PNS). This is followed by hematological & radiological investigations for those diagnosed for surgery. In addition to above, patients were given SNOTT-22 questionnaire preoperatively and NIPF using peak nasal inspiratory flow (PNIF) meter. Later, test was repeated at 2 month post-operatively.

## Inclusion criteria:

[1] All patients who were willing to participate.

[2] Those require corrective septal surgery for symptomatic DNS.

[3] All genders.

[4] 18 to 35 years.

## Exclusion criteria:

[1] Previous history of nasal septal surgeries.

[2] Those who have allergic rhinitis, chronic sinusitis with or without polyposis, bronchial asthma.

[3] Those who were suffering from nasal obstruction due to neoplastic etiology.

[4] Chronic smoker.

[5] Pregnancy.

## Statistical analysis:

SPSS v26 (IBM Corp.) in MS Excel spread sheet was used. Group comparisons for continuously distributed data were made using the independent sample (t) test when comparing two groups. Chi squared test was used for group comparisons for categorical data. In case the expected frequency in the conti ngency tables was found to be <5 for >25% of the cells, Fisher's exact test was used instead. Linear correlation between two continuous variables was explored using Pearson's correlation (where the data was normally distributed) and Spearman's correlation (for non normally distributed data). Statistical significance was kept at p < 0.05.

## Results:

Table 1 shows that, there were 23.5% patients between 18-21 years, 32.4% patients between 22-25 years and 26-30 years respectively and the remaining 11.8% patients between 31-35 years. On the other hand, 55.9% were male patients and 44.1% were female patients. Table 2 for NIPF showed that significant difference between pre-operative and post-operative NIPF scores (t= -19.626; p= .001).For SNOT 22 showed that significant difference between pre-operative and post-operative SNOT 22 scores (t= 8.244; p= .001) respectively. Table 3 showed a non-significant result indicating no significant change in NIPF and SNOT 22 scores over time between patients in different age groups. Table 4 shows that, non-significant result indicating no significant change in NIPF and SNOT 22 scores over time period between male and female patients.

## Discussion:

We have highlighted the need for an assessment tool post-surgery for DNS, as it is a common disorder encountered by Otorhinolaryngologists in routine practice. SNOT and NIPF was used to compare. Table 5 below mentioned different studies with different age & gender involvement compared to our study results.

## NIPF & SNOTT 22 score:

In our study, both SNOT 22 and NIPF scores improved postoperatively, suggesting better outcomes with septoplasty for DNS. The NIPF scores increased from 101.84 preoperatively to 120.15 postoperatively, suggesting better outcomes. Similarly, SNOT-22 scores reduced from 25.73 preoperatively to 6.05 postoperatively, showing a drastic improvement in nasal functioning. These findings were statistically significant as the p value was 0.001. Our study also showed a significant negative correlation between NIPF and SNOT 22 scores. Both tests gave similar results in measured outcomes for the patients post-surgery, in which patients felt improved symptoms and overall well-being. No significant differences in the scores between males and females or age existed as discussed in our Table 6 by comparing our study results with other results.

## Symptoms in SNOT-22 score:

Our study analyzed various parameters from the SNOT questionnaire. No significant changes in scores for dizziness, facial pain/pressure and sense of taste/smell were observed from the preoperative to the postoperative period. Additionally, factors such as the need to blow one's nose, sneezing, a runny nose, coughing, post nasal discharge, thick nasal discharge, ear fullness, ear pain/pressure, difficulty falling asleep and waking up at night, Feeling exhausted after a restless night, feeling tired throughout the day, decreased efficiency, decreased focus, feeling frustrated, restless, irritable, sad and embarrassed. The blockage and congestion of the nose showed significant improvements from before the surgery to after the surgery. In a study conducted by Sathish *et al.* several variables demonstrated significant improvement after surgery. These included symptoms such as the need to blow the nose, sneezing, running nose, nasal obstruction, loss of smell or taste, cough, post-nasal discharge, thick nasal discharge, ear fullness, facial pain/pressure, difficulty in falling asleep, waking up at night, lack of good night's sleep, waking up tired, reduced productivity and feeling embarrassed [10]. The findings of our study were nearly identical to these. In a recent study conducted by Abdelazim *et al.* notable enhancements were found in all sections of the questionnaire following nasal surgery [17]. People widely recognize septoplasty as the most effective treatment for deviated nasal septum (DNS). A notable advantageous outcome of the procedure is the reduction in systolic blood pressure, signifying a positive cardiovascular effect. Endoscopic septoplasty has shown positive results, requiring less tissue removal and enhancing the visualization of nasal structures. The choice to proceed with surgery should be based on a thorough assessment, using CT or CBCT scans, with a detailed review of the individual patient's risks and benefits [18]. Septoplasty is regarded as the most appropriate intervention for the correction of nasal septum deviation (NSD). The choice of a suitable technique is contingent upon the specific type of deviation and the unique characteristics of the individual. The surgical procedure, although regarded as advantageous with significant benefits, may result in unintended adverse effects. Post-operative complications, including bleeding and deformity, occur infrequently. Surgical interventions have demonstrated significant enhancements in patients' quality of life, resulting in high levels of patient satisfaction. Post-operative outcomes include favorable effects such as a reduction in systolic blood pressure, which has been noted as a beneficial cardiovascular side effect following the correction of NSD. The determination regarding the surgical intervention must be conducted following a thorough evaluation and cone beam computed tomography (CBCT), taking into consideration the specific risks associated with each patient [19]. The preoperative classification of septal deviations utilizing validated classification systems facilitates a more thorough evaluation of septal deviations, surpassing the mere identification of individual septal pathologies. So, the authors came to the conclusion that looking into the risk factors connected with revision septoplasty is important for finding complicated cases. This way, surgical techniques can be changed to improve each patient's outcomes, and patients can get better advice [20].

## Conclusion:

Data shows that both SNOT-22 and NIPF have shown significant negative correlation. Further, longitudinal cross sectional researches are encouraged to validate the results of our study.

## Figures and Tables

**Table 1:** Age & sex distribution

		**Frequency**	**Percent**
**Age groups**	18 - 21	8	23.5
**(in years)**	22 - 25	11	32.4
	26 - 30	11	32.4
	31 - 35	4	11.8
**Sex**	Male	19	55.9
	Female	15	44.1

**Table 2:** NIPF & SNOTT22 after the surgery

	**Time period**	**Mean**	**SD**	**Paired sample**
				**t-test**
**NIPF**	Pre-operative	101.8	3.78	t= -19.626
	Post-operative	120.2	3.88	p=.001
**SNOT 22**	Pre-operative	25.73	15.4	t= 8.244
	Post-operative	6.05	3.95	p=.001

**Table 3:** Age group difference

**Time**	**Age groups ( in years)**	**NIPF**		**SNOT 22**	
		**Mean**	**SD**	**Mean**	**SD**
**Pre-operative**	18- 21	101.8	3.2	20.5	10.6
	22 - 25	102.8	3.7	26.18	16.4
	26 - 30	100.8	4.5	31.45	18.6
	31 - 35	102.2	3.6	19.25	7.04
**Post-operative**	18- 21	119.5	3.6	5.5	3.29
	22 - 25	120.7	3.7	6.18	4.06
	26 - 30	120.8	4.7	6.45	5.18
	31 - 35	118.3	2.6	5.75	0.5
F-value (Time period*Age)		F=.607; p=.616		F=1.114; p=.359	

**Table 4:** Sex difference

**Time**	**Sex**	**NIPF**		**SNOT 22**	
		**Mean**	**SD**	**Mean**	**SD**
**Pre-operative**	Male	101.7	3.7	26.1	16.6
	Female	102.1	4.1	25.26	14.2
**Post-operative**	Male	120.1	4.5	6.84	4.53
	Female	120.2	3.1	5.06	2.91
F-value (Time period*Age)		F=.042; p=.838		F=.037; p=.849	

**Table 5:** Age & gender

**Authors**	**Age group**	**Mean age**	**Gender (n)**		**Total number**	**Name of the journal**	**Year of publication**
	**Maximum affected**		**Females (%)**	**Males (%)**			
Prakash *et al.* [9]	-	27.6	14	76	150	Nepalese Journal of ENT Head & Neck Surgery	2011
Satish *et al.* [10]	-	29.1	31.4	68.6	70	IOSR Journal of Dental and Medical Sciences	2013
Bugten *et al.* [11]	-	39	17.5	82.4	91	BMC Ear, Nose and Throat Disorders	2016
Khadgi *et al.* [12]	3rd decade		18.5	81.4	70	Kathmandu University Medical Journal	2021
Ottaviano *et al.*[13]	-	45	42.6	57.4	101	Journal of Personalized Medicine.	2022
Teixeira *et al.* [14]	-	-	60.3	39.7	78	Brazilian journal of otorhinolaryngology.	2011
Patel *et al.* [15]	-	30.9	35.7	64.3	28	European Archives of Oto-Rhino-Laryngology	2018
Our study	2nd to 3rd decade	-	44.1	55.9	34	--	-

**Table 6:** NIPF & SNOT score comparison

**Author**	**NIPF scores**					**SNOT scores**					**Journal**	**Year of publication**
	**Preoperative**	**Postoperative**			**P value**	**Preoperative**	**Postoperative**			**P value**		
		**1m**	**2 or 3m**	**6m**			**1m**	**2or 3 m**	**6m**			
Srinivasan *et al.* [16]	55	60	60	75	0.001	19.5	16	12	10	0.001	International Archives of Otorhinolaryngology	2021
Patel *et al.* [15]	79.8	101.4			< 0.0001						European Archives of Oto-Rhino-Laryngology	2018
Prakash *et al.* [9]						7.67	2.3			< 0.001	Nepalese Journal of ENT Head & Neck Surgery	2011
**(SNOT 10 was used)**												
Dizdar *et al.* [6]						38.3	20.45			0.227	Acta otorhinolaryngologica italica	2019
Sathish *et al.* [10]						26.93	17.01				IOSR Journal of Dental and Medical Sciences	2013
Our study	101.84	-	120.15	-	0.001	25.73	6.05			0.001	-	-

## References

[1] Ataol M, Ertas U (2020). International Journal of Pediatric Otorhinolaryngology..

[2] Illum P (1997). European archives of oto-rhino-laryngology..

[3] Berger G (2000). The laryngoscope..

[4] Berger G (2006). Archives of Otolaryngology-Head & Neck Surgery..

[5] Hatipoglu HG (2008). B-ENT..

[6] Dizdar D (2019). Acta Otorhinolaryngologica Italica..

[7] Kordjian HH, Schousboe LP (2017). Clinical Otolaryngology..

[8] Anderson ER (1999). Otolaryngology-Head and Neck Surgery..

[9] Prakash S (2011). Nepalese Journal of ENT Head & Neck Surgery..

[10] Satish HS (2013). IOSR J Dental Med Sci..

[11] Bugten V (2016). BMC Ear, Nose and Throat Disorders..

[12] Khadgi S (2021). Kathmandu University Medical Journal..

[13] Ottaviano G (2022). Journal of personalized medicine..

[14] Teixeira RU (2011). Brazilian journal of otorhinolaryngology..

[15] Patel B (2018). European Archives of Oto-Rhino-Laryngology..

[16] Srinivasan DG (2021). International Archives of Otorhinolaryngology..

[17] Abdelazim MH, Alsobky ME (2020). The Egyptian Journal of Otolaryngology..

[18] Ashwinirani SR (2024). Journal of Datta Meghe Institute of Medical Sciences University..

[19] Alghamdi FS (2022). Cureus..

[20] Bayer K (2024). Journal of Otorhinolaryngology, Hearing and Balance Medicine..

